# Antimicrobial Properties of Essential Oils Obtained from Autochthonous Aromatic Plants

**DOI:** 10.3390/ijerph20031657

**Published:** 2023-01-17

**Authors:** Francisco Ramiro Boy, María José Benito, María de Guía Córdoba, Alicia Rodríguez, Rocío Casquete

**Affiliations:** Nutrición y Bromatología, Instituto Universitario de Investigación en Recursos Agrarios (INURA), Escuela de Ingenierías Agrarias, Universidad de Extremadura, 06007 Badajoz, Spain

**Keywords:** essential oils, Dehesa plants, phenolic compounds, antimicrobial activity, aflatoxins

## Abstract

The aim of this work was to determine the antimicrobial activity of the essential oils of six plants widely distributed in the Dehesa of Extremadura, such as *Calendula officinalis*, *Cistus ladanifer*, *Cistus salviifolius*, *Cistus multiflorus*, *Lavandula stoechas*, and *Rosmarinus officinalis*. The content of total phenolic compounds (TPC) and the antimicrobial activity of the essential oils against pathogenic and spoilage bacteria and yeasts as well as aflatoxin-producing molds were determined. A great variability was observed in the composition of the essential oils obtained from the six aromatic plants. The *Cistus ladanifer* essential oil had the highest content of total phenols (287.32 ppm), followed by the *Cistus salviifolius* essential oil; and the *Rosmarinus officinalis* essential oil showed the lowest amount of these compounds. The essential oils showed inhibitory effects on the tested bacteria and also yeasts, showing a maximum inhibition diameter of 11.50 mm for *Salmonella choleraesuis* and *Kregervanrija fluxuum* in the case of *Cistus ladanifer* and a maximum diameter of 9 mm for *Bacillus cereus* and 9.50 mm for *Priceomyces carsonii* in the case of *Cistus salviifolius*. The results stated that antibacterial and antiyeast activity is influenced by the concentration and the plant material used for essential oil preparation. In molds, aflatoxin production was inhibited by all the essential oils, especially the essential oils of *Cistus ladanifer* and *Cistus salviifolius*. Therefore, it can be concluded that the essential oils of native plants have significant antimicrobial properties against pathogenic and spoilage microorganisms, so they could be studied for their use in the industry as they are cheap, available, and non-toxic plants that favor the sustainability of the environment of the Dehesa of Extremeña.

## 1. Introduction

The use in the pharmaceutical and food industry of medicinal and aromatic plants is recognized throughout the world [1], and these plants are the source of a wide variety of chemical compounds that are synthesized to perform important biological functions for the plants themselves [2]. In the Dehesa of Extremadura, many species of plants considered to be medicinal can be encountered, being relevant in the pharmaceutical sector [3,4]. Some of these medicinal plants include *Rosmarinus officinalis*, *Lavandula stoechas* [5] and *Cistus ladanifer*, *Cistus multiflorus*, and *Cistus salviifolius* [6]. Besides their medicinal properties, these are aromatic plants, which has made them excellent condiments for foods for many years. One of the most important benefits of these plants is their antimicrobial activity, and it has been shown that this is mainly because of the phenolic compounds contained in their flowers, stems, leaves, seeds, and fruits [7,8].

To guarantee the microbial safety of food, consumers are demanding “healthier” and eco-friendly food production systems, encouraging the development of novel biopreservation strategies based on the use of natural antimicrobial agents in place of synthetic preservatives. These concerns and the growing demand for organic foods are driving an increasing interest in natural antimicrobials, which show an effective antagonistic effect against a wide range of undesirable microorganisms in food. According to different researchers, the growth of pathogenic and spoilage microorganisms can be strongly reduced or inhibited by several plant extracts, some of these plants being native to the Dehesa of Extremadura [7,9]. Phenolic compounds seem to be the main substances responsible for the antimicrobial effect, and this has been mainly associated with the presence of hydroxyl groups in their molecules. In fact, the position and number of these groups, i.e., the hydroxylation pattern, on the phenolic ring appears to be linked to the importance of the inhibitory properties exerted by phenolic compounds on microorganisms [10,11]. Thus, a bactericidal effect has been demonstrated against food-borne bacteria, such as *Listeria monocytogenes, Staphylococcus aureus*, *Salmonella* sp., and *Escherichia coli* [12,13,14]; spoilage yeasts [9]; and food pathogenic molds [15].

Most of the natural alternatives to synthetic food preservatives explored in recent studies are plant extracts in crude or purified form, mainly essential oils or pure compounds, most of which have been used since ancient times. These have become the focus of interest for direct application in food products [16]. Essential oils are the plant extracts that have been most studied for their antimicrobial activity.

Essential oils are aromatic oily liquids obtained from plant material (flowers, buds, seeds, leaves, twigs, bark, herbs, wood, fruits, and roots). They can be obtained by different methods, but the steam distillation method is most commonly used for the commercial production of essential oils [17]. Other extraction methods, such as extraction using liquid carbon dioxide at low temperature and high pressure, produce a more natural compound and organoleptic profile, but this is much more expensive and laborious [18]. For this reason, hydrodistillation is the most used method, although the high extraction temperature may cause some volatile components to be lost and this may also influence antimicrobial properties. This seems to be confirmed by the fact that herbal essential oils extracted with hexane have been shown to exhibit higher antimicrobial activity than the corresponding steam-distilled essential oils [19]. Essential oils are volatile and should therefore be stored in airtight containers and in the dark to avoid changes in their composition, as they are susceptible to damage by factors such as light, heat, oxidation, and hydration.

Essential oils may have more than 60 individual components, depending on the species and subspecies of the plant from which they are obtained, and the major components may constitute up to 85% of an essential oil, while other components are present only in trace form [18]. They contain cyclic and acyclic compounds of different types, such as alcohols, esters, phenols, ketones, lactones, aldehydes, and oxides. According to different authors, phenolic compounds are mainly responsible for antibacterial properties, as mentioned before; however, there is evidence that minor compounds play a key role in antibacterial activity, possibly by producing a synergistic effect among other compounds [20]. It is therefore logical to think that considering the great variety of chemical compounds present in essential oils, their antimicrobial activity is not attributable to a specific mechanism of action but to the combined action of several of them on different parts of the microbial cell.

Essential oils are effective against molds, yeasts, and bacteria; however, different levels of susceptibility have been observed for each of group probably due to the difference in their membranes, as occurs with Gram-negative and Gram-positive bacteria [21]. There are also different studies on the inhibition of mold growth; however, there are not so many on the influence on mycotoxin production [22].

For this reason, although there are a large number of studies on essential oils obtained from aromatic plants, there are few reports focused on native plants of the Dehesa of Extremeña Therefore, this study aims to evaluate essential oils obtained from different native plants of the Dehesa of Extremeña, which have been scarcely studied, for their antimicrobial activity against pathogenic bacteria, spoilage yeasts, and aflatoxin-producing molds.

## 2. Materials and Methods

### 2.1. Plant Material and Essential Oil Preparation

For the development of this work, different parts, as specified by Boy et al. [9], of 6 plant species (flowers from *Calendula officinalis*; stems from *Cistus ladanifer, Cistus multiflorus, Cistus salviifolius,* and *Lavandula stoechas*; and leaves from *Rosmarinus officinalis*) were used. They were collected in the region of Extremadura (Spain) by a local company in February 2021. The samples were packed in plastic bags under vacuum and stored at −20 °C until they were used to obtain the essential oils. The essential oils were obtained by steam distillation of the aerial parts, flowers, stems, and leaves of wild plants. The essential oils were obtained according to the procedure described in the European Pharmacopoeia, using 100 g of dried plant material, completely immersed in distilled water and subjected to hydrodistillation for 3 h, using a Clevenger-type apparatus, in which the essential oils were obtained as final products. All essential oils were stored in dark glass bottles at 4 °C until further testing.

### 2.2. Bacterial, Yeasts, and Mold Strains

Pathogenic and spoilage food-borne bacteria, yeasts, and molds in food were used to carry out the study. The pathogenic bacteria were *Staphylococcus aureus* CECT 976, *Bacillus cereus* CECT 131, *Listeria monocytogenes* CECT 911, *Listeria innocua* CECT 910, *Salmonella choleraesuis* CECT 4395, and *Escherichia coli* CECT 4267, which were obtained from the Spanish Type Culture Collection (CECT). For spoilage yeasts, *Candida boidinii* CECT 11153, *Priceomyces carsonii* CECT 10230, *Kregervanrija fluxuum* CECT 12787, and *Zygosacharomyces bailii* CECT 11043 were used, also collected from the Spanish Type Culture Collection (CECT). Finally, 2 strains of *Aspergillus flavus* strains 1 and strain 2 (CQ8 and CQ103) that produce aflatoxins B_1_ and B_1_ isolated from plants [23] were used.

### 2.3. Total Phenolic Content

The total phenolic content was determined using Folin–Ciocalteu reagent according to the method described by Wettasinghe and Shahidi [24] in a UV-1800 spectrophotometer (Shimadzu Scientific Instruments, Columbia, MD, USA). As a standard, gallic acid was used. Results are expressed in mg/L. All experiments were performed in triplicate.

### 2.4. Antimicrobial Activity against Bacteria and Yeasts

Suspensions of target cells (Section 2.2) were obtained from cultures incubated overnight at 37 and 25 °C on brain heart infusion agar (BHI; Oxoid, Madrid, Spain) for bacteria and peptone agar and dextrose extract (YPD; Oxoid, Madrid, Spain) for yeasts. Strains were transferred to a peptone water sterile solution (Scharlab, Barcelona, Spain) to reach a turbidity level equivalent to 0.5 McFarland standards after the incubation time had been completed. Next, 1 mL of each suspension was pipetted into separate sterile Petri dishes, to which was added 20 mL of melted (45 °C) BHI for bacteria and YPD for yeasts with 1% agar. Plates with 10 µL of each essential oil were spread over the surface of the plate. The plates were incubated at 37 °C for bacteria and at 25 °C for yeasts overnight, and the resulting diameter (mm) of the inhibition zone was measuring. All experiments were performed in triplicate.

### 2.5. Antimicrobial Activity against Aspergillus Flavus Strains

First, inocula of the two *Aspergillus flavus* strains were prepared. For this purpose, the strains were initially grown on malt extract agar (Scharlab S.L., Barcelona, Spain) at 25 °C for 10 days. A spore suspension of each strain was collected by adding 10 mL of sterile 0.05% (vol/vol) Tween 80 (Scharlab S.L.) to each mold plate and then rubbing the surface with a glass rod. The suspension formed was filtered through 2 layers of gauze. The concentration of each spore suspension was quantified using a Neubauer chamber and a microscope (Olympus CX 400, Tokyo, Japan) and adjusted to 10^6^ spores/mL with sterile water. 

For antifungal activity, potato dextrose agar (PDA; Oxoid, Madrid, Spain) plates were prepared and 100 μL of each essential oil was added and spread over the surface of the plate. After spreading, the plates were allowed to dry under sterile conditions around a flame.

For the assay, 10 μL of the spore suspension of each mold was inoculated in a central spot of the plate. The strains were inoculated separately. Sterile water was used as a negative control. Plates were incubated at 25 °C for 10 days.

#### 2.5.1. Assessment of Growth 

The diameter of the growing colonies was measured in 2 perpendicular directions every 24 h during the 10 days. The average of both diameters was recorded as the growth measurement for each strain. The mean colony diameter (mm) of each experiment and condition was plotted against incubation time (days) to establish growth curves for each fungal strain, as described by Casquete et al. [23]. The colony growth rate (μ (mm/days)) was determined from the slope of the growth curve.

#### 2.5.2. Aflatoxin Determination

Mycelia were harvested at 10 days, and five 4-mm-diameter agar plugs were extracted from each fungal colony culture in 2 mL microcentrifuge tubes. For extraction for aflatoxin quantification, the procedure of Casquete et al. [23] was followed. Aflatoxin analysis was performed with an Agilent 1100 series HPLC system (Agilent Technologies, Santa Clara, CA, USA) equipped with a diode array detector (Agilent G1315B) set at 360 nm and using a C18 HPLC column (250 × 4.6 mm, 5 μm particle size; Supelco, Bellefonte, PA, USA). A calibration curve was plotted for aflatoxins B_1_ and B_2_ (Sigma Chemical Co., St. Louis, MO, USA). Results are expressed as ppb.

### 2.6. Statistical Analysis

Statistical processing of data was performed using SPSS for Windows, version 21.0 (SPSS Inc., Chicago, IL, USA). Statistical descriptions of the results were established, and differences, both between and within groups, were analyzed using one-way analysis of variance (ANOVA) and explained using Tukey’s honest significant difference test (*p* < 0.05). 

## 3. Results and Discussion

The total phenolic compounds of the essential oils obtained from the different plants used in the study are presented in Table 1.

In general, it was observed that the *Cistus ladanifer* essential oil showed the highest amount of phenolic compounds, 287.32 mg/L, followed by the *Cistus salviifolius* essential oil; and the *Rosmarinus officinalis* essential oil showed the lowest amount of these compounds.

Boy et al. [9] studied the extraction of phenolic compounds from these plants using two different methods of agitation and ultrasound and found that *Cistus ladanifer* and *Cistus salviiflolius* stems show the highest amounts of phenolic compounds using the two extraction methods compared to *Calendula officinalis* and *Rosmarinus officinalis*. However, *Cistus multiflorus* and *Lavandula stoechas* also showed high amounts of phenolic compounds using these two methods in contrast to those in the essential oils of this study. The extraction method, therefore, significantly influences the composition of phenolic compounds, as suggested by different researchers [25].

Table 2 shows the antimicrobial effect of the essential oils studied on the six pathogenic bacteria analyzed: *Listeria monocytogenes*, *Listeria innocua*, *Staphylococcus aureus*, *Bacillus cereus*, *Escherichia coli*, and *Salmonella choleraesuis*.

It can be observed that *Cistus ladanifer* and *Cistus salviifolius* essential oils presented a greater inhibition capacity on the six pathogenic bacteria studied (*p* < 0.05), showing a maximum inhibition diameter of 11.50 mm for *Salmonella choleraesuis* in the case of *Cistus ladanifer* and a maximum diameter of 9 mm for *Bacillus cereus* in the case of *Cistus salviifolius*. The *Lavandula stoechas* essential oil only showed a greater inhibition capacity against *Listeria innocua*—7.50 mm as the diameter of inhibition. The other three essential oils, from *Calendula officinalis*, *Cistus multiflorus*, and *Rosmarinus officinalis*, did not show activity against any of the pathogenic bacteria studied.

These results agree partially with the essential oils that contained a higher concentration of phenolic compounds, which were those of *Cistus ladanifer* and *Cistus salviiflolius*. However, this did not occur in the case of *Lavandula stoechas*, which presented significant activity against *Listeria innocua* and had a low concentration of phenolic compounds in the essential oil. Consequently, this supports the fact that not only the concentration of phenolic compounds present in the essential oil is important but also the nature of the compounds present and their synergies [20]. Gram-positive bacteria are known to be more sensitive to natural extracts, and Gram-negative bacteria are reported to be less sensitive [21]. However, exceptions have been reported in which Gram-negative bacteria were found to be more susceptible than Gram-positive bacteria [9,26,27], highlighting the susceptibility of *Salmonella cholerasuis* and *Escherichia coli*. In contrast, the genus *Cistus* has been remarked by some authors for its antimicrobial capacity, after testing the antimicrobial activity of different species of the genus *Cistus* and observing greater activity against diverse pathogenic bacterial species and higher activity against Gram-negative ones [9,27]. The essential oil obtained from *Cistus multiflorus* did not show activity against any of the evaluated bacteria, which is in agreement with the work of other authors [9]. *Calendula officinalis* and *Rosmarinus officinalis* essential oils also showed no activity, although the antimicrobial effects of these plant extracts against bacteria have been reported at high concentrations [28,29].

According to the results, it is possible to conclude that the antibacterial activity may be influenced by the concentration and the plant material used for essential oil preparation.

The antimicrobial effect of the essential oils on the four spoilage yeasts was also studied (Table 3).

In this case, *Cistus ladanifer* and *Cistus salviifolius* essential oils also showed the greatest inhibitory capacity against the four yeasts studied, with an inhibition diameter between 11.50 and 10 mm for *Cistus ladanifer* and 9.50 and 7.50 mm for *Cistus salviifolius*, according to the greater concentration of phenolic compounds in these essential oils. However, the *Calendula officinalis* essential oil was active against the yeasts *Kregervanrija fluxuum*, with an inhibition diameter of 7.50 mm, and *Priceomyces carsonii*, with an inhibition diameter of 10.50 mm, and this plant’s essential oil had a low concentration of phenolic compounds. These results are in agreement with those obtained previously in studies with extracts obtained from *Cistus* plants.

Comparing the results presented in Table 2 and Table 3, it was observed that the essential oil of *Calendula officinalis* did not show activity against bacteria but did show activity against yeasts in contrast with the essential oil of *Lavandula stoechas*. Therefore, as in the case of antibacterial activity, the activity against yeasts is affected by the concentration of phenolic compounds as well as by the plant material used for essential oil preparation.

There are different studies in the bibliography that highlight the relevance of the extracts and oils of *Lavandula stoechas* and *Rosmarinus officinalis* for yeasts [30,31]; however, as previously mentioned in the case of bacteria, the activity takes place at high concentrations of phenolic compounds.

**Figure 1:**
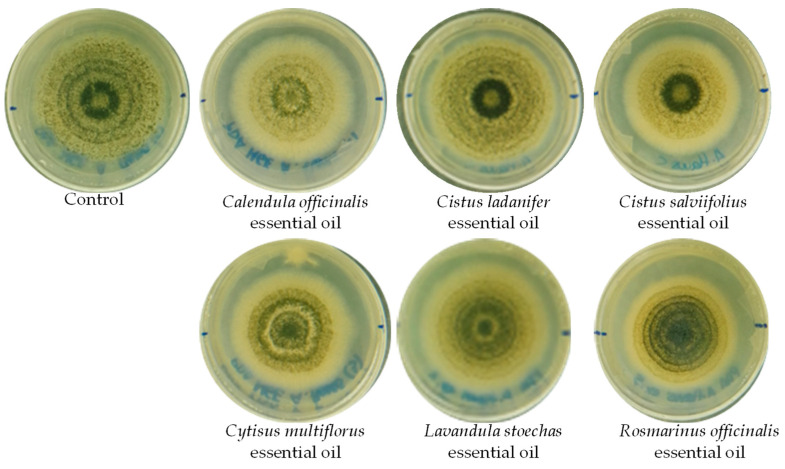
Growth of *Aspergillus flavus* strain 1 in PDA medium after 10 days of incubation in the presence of different essential oils.

Finally, the essential oils were tested for their influence on the growth of two strains of *Aspergillus flavus* and their effect on aflatoxin production. This assay was carried out for 10 days of incubation at 25 °C. Figure 1 shows the final growth of strain 1 in PDA medium in the presence of the different essential oils after 10 days of incubation and the control without the addition of any essential oil. It can be observed that at the end of the study, *A. flavus* grew with a similar growth rate in the presence of the essential oils tested. This also was observed in the case of strain 2. The final growths were between 4 and 3.3 cm diameter at 10 days for strain 1 and between 4 and 5 cm diameter for strain 2.

Regarding the growth rate results, both strains of *Aspergillus flavus* grew at a slower rate in the presence of the essential oils and significantly in the presence of *Cistus multiflorus* and *Rosmarinus officinalis* essential oils for strain 1 and in the presence of all essential oils except *Lavandula stoechas* for strain 2 (Table 4). 

Although the essential oils assayed showed a similar effect on *Aspergillus flavus* growth, significant differences were observed in aflatoxin production. For aflatoxin B_1_ (AFB_1_) production, the essential oils of *Cistus ladanifer* and *Cistus salviiflolius* had the greatest effect, followed by the essential oils of *Cistus multiflorus*, *Lavandula stoechas*, *Rosmarinus officinalis*, and, finally, *Calendula officinalis* (Table 5).

The production of aflatoxin B_2_ (AFB_2_) in both strains was lower than that of AFB_1_. Although significant differences were observed with respect to the control, for AFB_2_, no differences were detected with respect to the effect of the different essential oils, probably because as less aflatoxin is produced, the impact may be more difficult to evaluate.

There are different studies highlighting the ability of plant essential oils to inhibit the growth of molds. Wan et al. [22] investigated the effect of the nanoemulsions of selected essential oils (thyme, lemongrass, cinnamon, peppermint, and clove) on antifungal activity and mycotoxin inhibitory activity using two strains of *Fusarium graminearum*. Regarding the inhibition of mycotoxin production, significant inhibitory activity was observed and the same essential oils presented significant differences in the inhibition of mycotoxin production in the two strains of *Fusarium graminearum*, which coincides with our results, since we also obtained differences in the inhibition of the two strains by the same essential oils. The antifungal activity of widely known essential oils, such as oregano (*Origanum vulgare*) [32], cinnamon [33], clove [34], peppermint, and eucalyptus [35] has also been tested.

Few research works have focused on the impact of essential oils on mycotoxin production, and widely available native plants, such as those of the genus *Cistus*, as in this work, have proved to be the ones with the greatest effect.

## 4. Conclusions

In general, when the different essential oils used were compared, it was observed that *Cistus ladanifer* and *Cistus salviifolius* essential oils had the highest amount of phenolic compounds, and this may be one of the reasons why these essential oils showed higher antimicrobial activity against all the microorganisms used, including bacteria, yeasts, and molds. However, against the pathogenic bacteria tested, the essential oil of *Lavandula stoechas* showed significant activity against *Listeria innocua* and had a low concentration of phenolic compounds. Something similar occurred with the essential oil of *Calendula officinalis*, which was also active against two of the yeasts tested (*Kregervanrija fluxuum* and *Priceomyces carsonii*), although this essential oil also had a low concentration of phenolic compounds. In the case of experiments on the inhibition of *Aspergillus flavus* growth and aflatoxin production, all the essential oils inhibited production, especially the essential oils of *Cistus ladanifer* and *Cistus salviifolius*, followed by the other four essential oils. Thus, the activities observed were different in the assays, depending on the microorganisms studied, which corroborates the fact that microorganisms are affected by the concentration of phenolic compounds as well as by the plant material used for the preparation of the essential oil.

Therefore, it can be concluded that the essential oils of native plants have significant antimicrobial properties against pathogenic and spoilage microorganisms, so they could be studied for their use in the industry as they are cheap, available, and non-toxic plants that favor the sustainability of the environment of the Dehesa of Extremeña. However, future analysis of the specific chemical compounds occurring in these essential oils should be carried out using gas chromatography–mass spectrometry (GC-MS).

## Figures and Tables

**Table 1:** Total phenolic compounds (mg/L) of essential oils obtained from aromatic plants.

Essential Oil	Total Phenolic Content (mg/L)		
	Mean		SD ^1^
*Calendula officinalis*	54.26	±	0.77 ^c^
*Cistus ladanifer*	287.32	±	8.10 ^a^
*Cistus salviifolius*	228.64	±	16.36 ^b^
*Cistus multiflorus*	40.68	±	7.05 ^c^
*Lavandula stoechas*	44.37	±	1.81 ^c^
*Rosmarinus officinalis*	10.66	±	5.25 ^d^

^1^ SD: standard deviation. ^a–d^ Values with different superscript letters are significantly different (*p* < 0.05) between plants.

**Table 2:** Activity of different essential oils obtained from aromatic plants against pathogenic bacteria, expressed as the diameter of inhibition zones in mm.

Essential Oil	*Listeria* *monocytogenes*			*Listeria* *innocua*			*Staphylococcus* *aureus*			*Bacillus* *cereus*			*Escherichia* *coli*			*Salmonella* *choleraesuis*		
	Mean		SD ^1^	Mean		SD	Mean		SD	Mean		SD	Mean		SD	Mean		SD
*Calendula officinalis*	-			-			-			-			-			-		
*Cistus ladanifer*	9.50	±	0.71 ^a^	9.50	±	0.71 ^a^	8.00	±	0.24 ^a^	9.00	±	1.51 ^a^	10.00	±	0.00 ^a^	11.50	±	0.71 ^a^
*Cistus salviifolius*	3.50	±	4.95 ^b^	7.50	±	0.71 ^b^	6.75	±	0.35 ^b^	9.00	±	3.24 ^a^	7.50	±	0.71 ^b^	6.50	±	0.71 ^b^
*Cistus multiflorus*	-			-			-			-			-			-		
*Lavandula stoechas*	-			7.50	±	0.71 ^b^	-			-			-			-		
*Rosmarinus officinalis*	-			-			-			-			-			-		

^1^ SD: standard deviation; -: no inhibition; ^a,b^: statistical differences (*p* < 0.05) found between the values are indicated by different superscript letters.

**Table 3:** Activity of different essential oils obtained from aromatic plants against yeasts, expressed as the diameter of inhibition zones in mm.

Essential Oil	*Candida* *boidinii*			*Kregervanrija* *fluxuum*			*Priceomyces* *carsonii*			*Zygosacharomyces* *bailii*		
	Mean		SD ^1^	Mean		SD	Mean		SD	Mean		SD
*Calendula officinalis*	-			7.50	±	0.71 ^b^	10.50	±	0.71 ^a^	-		
*Cistus ladanifer*	10.50	±	0.71 ^a^	11.50	±	0.71 ^a^	10.50	±	0.71 ^a^	10.00	±	0.00 ^a^
*Cistus salviifolius*	7.50	±	0.71 ^b^	7.50	±	0.71 ^b^	9.50	±	0.71 ^a^	7.50	±	0.71 ^b^
*Cytisus multiflorus*	-			-			-			-		
*Lavandula stoechas*	-			-			-			-		
*Rosmarinus officinalis*	-			-			-			-		

^1^ SD: standard deviation; -: no inhibition; ^a,b^: statistical differences (*p* < 0.05) found between the values are indicated by different superscript letters.

**Table 4:** Growth rate (mm/day) of *A. flavus* strains 1 and 2 that produce aflatoxins B_1_ and B_2_ obtained over 10 days of incubation at 25 °C.

Essential Oil	*A. flavus* Strain 1			*A. flavus* Strain 2		
	Mean		SD ^1^	Mean		SD
Control	4.07	±	0.15 ^a^	5.03	±	0.35 ^a^
*Calendula officinalis*	3.68	±	0.26 ^a,b^	4.32	±	0.19 ^b^
*Cistus ladanifer*	3.83	±	0.13 ^a,b^	4.48	±	0.22 ^b^
*Cistus salviifolius*	3.73	±	0.10 ^a,b^	4.26	±	0.09 ^b^
*Cistus multiflorus*	3.38	±	0.03 ^b^	4.04	±	0.37 ^b^
*Lavandula stoechas*	3.69	±	0.12 ^a,b^	4.81	±	0.25 ^a,b^
*Rosmarinus officinalis*	3.42	±	0.03 ^b^	4.16	±	0.10 ^b^

^1^ SD: standard deviation; ^a,b^: values with different superscript letters in the same column indicate statistical differences (*p* < 0.05).

**Table 5:** Production of aflatoxins B_1_ and B_2_ (ppb) of *Aspergillus flavus* strains 1 and 2 determined in samples with different essential oils at the end of incubation.

Essential Oil	*Aspergillus flavus* Strain 1						*Aspergillus flavus* Strain 2					
	Aflatoxin B1			Aflatoxin B2			Aflatoxin B1			Aflatoxin B2		
	Mean		SD ^1^	Mean		SD	Mean		SD	Mean		SD
Control	1406.93	±	406.06 ^a^	77.60	±	12.15 ^a^	750.28	±	406.06 ^a^	78.34	±	11.08 ^a^
*Calendula officinalis*	516.91	±	227.43 ^b^	7.80	±	1.26 ^b^	636.55	±	227.43 ^b^	16.01	±	9.45 ^b^
*Cistus ladanifer*	118.35	±	48.96 ^c^	15.23	±	7.03 ^b^	259.75	±	48.96 ^c^	10.23	±	4.89 ^b^
*Cistus salviifolius*	164.94	±	68.31 ^c^	1.70	±	0.02 ^c^	129.71	±	68.31^c^	2.92	±	0.36 ^b^
*Cistus multiflorus*	309.78	±	52.93 ^b^	5.65	±	1.26 ^b^	342.46	±	52.93 ^b,c^	4.61	±	1.20 ^b^
*Lavandula stoechas*	313.00	±	45.29 ^b^	10.02	±	1.26 ^b^	335.63	±	45.29 ^b,c^	8.36	±	4.12 ^b^
*Rosmarinus officinalis*	268.47	±	84.90 ^b,c^	9.98	±	2.10 ^b^	393.61	±	84.90 ^b,c^	9.98	±	2.10 ^b^

^1^ SD: standard deviation; ^a–c^: values with different superscript letters in the same column indicate statistical differences (*p* < 0.05).

## Data Availability

Not applicable.
